# Paternal DEHP Exposure Triggers Reproductive Toxicity in Offspring via Epigenetic Modification of H3K27me3

**DOI:** 10.3390/toxics13030172

**Published:** 2025-02-27

**Authors:** Lu Zhang, Rui Yang, Guiyong Xu, Lingqiao Wang, Weiyan Chen, Yao Tan, Guowei Zhang, Wenbin Liu, Guanghui Zhang, Jun Li, Ziyuan Zhou

**Affiliations:** 1 The Key Laboratory of Environmental Pollution Monitoring and Disease Control, School of Public Heath, Ministry of Education, Guizhou Medical University, Guiyang 561113, China; zl1332271608@163.com (L.Z.); yangr202501@163.com (R.Y.); xuguiyong73@163.com (G.X.); 2 Department of Environmental Health, College of Preventive Medicine, Third Military Medical University (Army Medical University), Chongqing 400038, China; mamababa520qq@163.com (L.W.); weiyanchen@tmmu.edu.cn (W.C.); xiaoyue7122@tmmu.edu.cn (Y.T.); zhangguowei@tmmu.edu.cn (G.Z.); liuwenbin@tmmu.edu.cn (W.L.); zhgh221@tmmu.edu.cn (G.Z.)

**Keywords:** DEHP, paternal exposure, male offspring effect, H3K27me3, apoptosis

## Abstract

Di (2-ethylhexyl) phthalate (DEHP) is an acknowledged endocrine disruptor with male reproductive toxicity; nevertheless, the transgenerational impacts on male offspring resulting from paternal exposure, along with the mechanisms involved, are not well understood. To develop a transgenerational model of DEHP paternal exposure, male C57BL/6J mice (4-week) exposed to DEHP (5, 250, and 500 mg/kg/d) for 35 days were then bred with unexposed female mice at a ratio of 1:2 to produce offspring. Findings indicate that the sperm quality and relative sex hormones were adversely affected in males of F1 and F2 generations, and pathological damage in the testes and the apoptosis of testicular cells were also observed. Interestingly, an increase in the expression levels of H3K27me3 was observed in the testicular tissues of male descendants. It was further confirmed by in vitro approach that H3K27me3 may down-regulate the expression of Bcl-2 and plays a role in regulating the initiation of apoptosis in Leydig cells triggered by MEHP (the primary metabolite of DEHP). Additionally, the down-regulation of Bcl-2 can be reversed by treatment with the H3K27me3 inhibitor GSK126. To conclude, DEHP leads to transgenerational harm to male offspring reproductive systems, with the epigenetic mechanism of H3K27me3 playing a key role in mediating these effects.

## 1. Introduction

In recent years, global male semen concentrations and total sperm counts have declined by more than 50% [1]. At the same time, studies have shown that environmental pollution can cause damage to sperm, which is an early warning sentinel of human health [2]. Environmental pollution, including heavy metals [3], environmental endocrine disruptors [4], and persistent organic pollutants [5], can cause adverse effects on the male reproductive system, such as reduced sperm quality and pathologic changes in gonadal tissue, which may increase the risk of infertility. Consequently, male reproductive toxicity has already become a significant public concern. Exposure to environmental endocrine disruptors is among the most critical causes of male reproductive harm [6]. DEHP is one of these, and it is abundant in the environment because it is unstable in bonding with plastics and easily escapes into the environment [7]. Research has found that DEHP resulted in a reduction in relative testicular weight and serum testosterone levels in male mice, with sperm quality also being adversely affected, ultimately leading to decreased fertility [8]. Similarly, exposure of adolescent male mice to DEHP at doses of 500 mg/kg/d was found to down-regulate histone methylation levels and induce reproductive disorders, including germ cell apoptosis and reduced testosterone levels [9]. Furthermore, DEHP entering the mother causes hypermethylation of spermatogenesis-associated genes in the F1 generation of male mice, resulting in down-regulation of gene expression and spermatogenesis defects [10]. The experimental studies described above emphasize the risk of reproductive and developmental toxicity of DEHP in males and its potential to negatively affect offspring.

Research evidence indicates that parental lifestyle [11], diet [12], and exposure to endocrine disruptors [13] may be transmitted to offspring through epigenetic modifications, and that offspring may exhibit adverse phenotypes, including abnormal embryonic development, altered reproductive endocrinology, and changes in growth and development. DEHP is recognized as epigenotoxic [14]. DEHP-induced intergenerational and transgenerational epigenetic effects can be transmitted through epigenetic mechanisms including DNA methylation [15], small noncoding RNAs [16], or histone modifications [17], particularly through histone modifications. Histone H3K27 trimethylation, a conserved histone modification, represses gene expression and serves as an epigenetic mechanism to regulate transgenerational genetic effects in Caenorhabditis elegans [18], Drosophila melanogaster [19], and mice [20].

Given that the fetus develops within the maternal environment, it is particularly susceptible to intrauterine exposure [21], and previous research has focused on the effects of maternal DEHP exposure on pregnancy outcomes, as well as the growth and development of the offspring. Recently, it has been demonstrated that DEHP causes not only male reproductive damage but also adverse phenotypes in their offspring, but the reasons for these alterations still remain unclear [22]. Overall, in this research we aim to elucidate the adverse effects of paternal DEHP exposure on male offspring by establishing a transgenerational animal model. Furthermore, we seek to investigate the potential involvement of the histone modification H3K27me3 in the transgenerational effects induced by DEHP.

## 2. Materials and Methods

### 2.1. Animal Model

**Figure 1:**
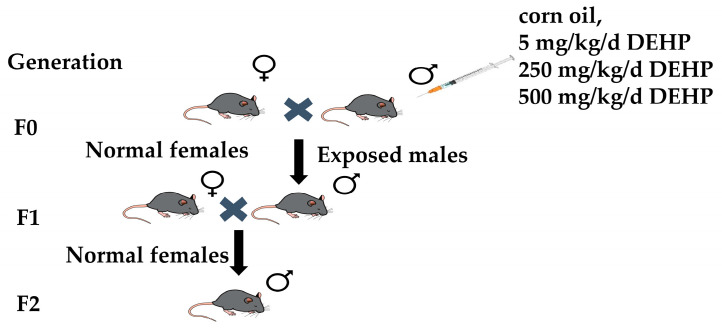
Schematic diagram of cross-generation model construction for paternal DEHP exposure.

Forty C57BL/6J male mice (Beijing Vital River Laboratory Animal Technology Co., Ltd., Beijing, China), 3 weeks old and weighing 13–16 g, were housed in a standard environment and given adequate water and food. After one week, they were divided into control (corn oil) and DEHP-contaminated groups (5, 250, and 500 mg/kg/d) of 10 mice each according to the randomized numeric table method (Figure 1). Corn oil and DEHP were purchased from Sigma-Aldrich (St. Louis, MI, USA). Gavage was performed at the same time every day for 35 days (one spermatogenic cycle) based on body weight. All animal studies were authorized by the Laboratory Animal Welfare and Ethical Committee of the Army Medical University (AMUWEC20226243).

### 2.2. Reproduction and Culture of Offspring

As shown in Figure 1, male mice were directly exposed to DEHP and mated (1:2) with DEHP-unexposed female mice to breed the F1 generation; then, male mice of the F1 generation were mated (1:2) with unexposed female mice to produce the F2 generation. The F1 generation is called intergenerational and the F2 generation is called transgenerational.

### 2.3. Anogenital Distance (AGD) and Prepuce Separation Test

In male offspring, at postnatal days 14, 19, and 24 (PND14, PND19, and PND24), the anogenital distance was measured using vernier calipers and their body weights were recorded [23]. Starting from PND21, male mice were observed daily for foreskin separation, and the time of foreskin separation and body weight were recorded.

### 2.4. Organization Sample Collection

Male mice were weighed and bled in the eyeballs and then severed and executed, and the abdomen was cut open to rapidly separate the testes and epididymis bilaterally, which were weighed and recorded (testis coefficient = bilateral testis weight/body weight × 100%; epididymis coefficient = bilateral epididymis weight/body weight × 100%). Some organs were fixed with 4% paraformaldehyde, and the rest were stored at −80 °C. Determination of reproductive hormone levels in the serum of male offspring was conducted according to the instructions of the mouse testosterone ELISA kit, mouse LH ELISA kit, mouse FSH ELISA kit, and mouse ABP ELISA kit (Weiao Biotechnology, Shanghai, China).

### 2.5. Sperm Quality Assessment

Semen parameters of male offspring were examined by using a computer-aided sperm analysis (CASA) system. Isolated epididymal tails were placed in 1 mL of medium containing BSA at 37 °C and incubated, immediately following the addition of 10 μL of sperm-containing culture solution on a disposable sperm counting plate, and the concentration and viability were assessed using the CASA system, with a minimum of 500 spermatozoa per mouse used for data analysis.

### 2.6. Testicular Pathologic Histologic Analysis

**Figure 2:**
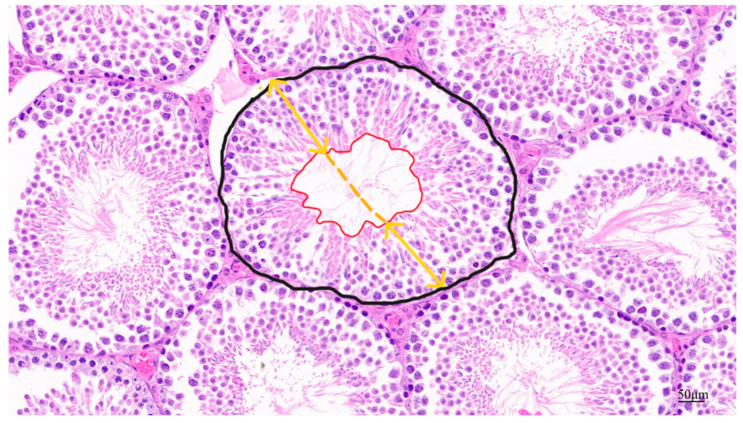
Schematic diagram of parameters for quantitative assessment of testicular tissue in male mice.

Fixed testicular tissue slides were stained with hematoxylin and eosin and photographed under light microscopy. Testicular tissue lumen area, seminiferous tubular area, epithelial height, and diameter were assessed. The black line area shown in Figure 2 indicates the cross-sectional area of the spermatogenic tubules and the red line area is the lumen area. The yellow line represents the vertical diameter of the varicocele, and the average length of the solid portion of the line represents the height of the varicocele epithelium [24]. Apoptosis in testicular paraffin sections was identified using a TUNEL staining kit (Servicebio, Wuhan, China) according to the instructions and the sections were analyzed using confocal fluorescence microscopy.

### 2.7. Cell Processing

Mouse testicular mesenchymal cells TM3 were purchased from Timesaver (Shanghai, China) and cultured in F12 medium (Sigam, Newbury, UK) containing 5% fetal bovine serum (FBS (fetal bovine serum), Hyclone, Logan, UT, USA). Cells treated with MEHP (100, 200, and 400 μM) were incubated at 37 °C for 24 h in an incubator with 5% CO_2_.

### 2.8. Apoptosis Assay

Based on the experimental procedures, we employed the Annexin V-FITC kit (BD Pharmingen, Franklin Lakes, NJ, USA) to detect apoptosis. Cells treated with MEHP were incubated with Annexin V-FITC and propidium iodide (PI) for 15 min at room temperature, and protected from light. Subsequently, flow cytometry was utilized to carry out the assay. A total of 10,000 cells were collected from each sample and the apoptosis rate was calculated by flow cytometry based on the number of apoptotic cells identified by fluorescence (Apoptosis rate = number of apoptotic cells/10,000 × 100%).

### 2.9. Protein Extraction and Western Blot

Cells were lysed on ice with RIPA buffer for 30 min; testicular tissue was homogenized for 6 min, and then centrifuged for 10 min to extract the supernatant; and protein concentration was determined by BCA Protein Assay Kit (Biotronik, Beijing, China). The protein samples were separated by SDS-PAGE electrophoresis and then transferred to PVDF membranes (Millipore, Bedford, MA, USA). The membranes were incubated with anti-β-actin (rabbit, AC026), anti-TriMethyl-Histone H3-K27 (rabbit, A22396), anti-Bax (rabbit, A19684), and anti-Histone H3 (rabbit, A17562) from ABclonal (Wuhan, China), and anti-Bcl-2 (mouse, 68103-1-Ig) from Proteintech (Wuhan, China), at 4 °C overnight. After washing three times, the membrane was incubated with HRP coupled secondary antibody (1:2000) for 1 h and then the signals were detected using enhanced chemiluminescence (ECL) solution (Millipore, USA).

### 2.10. Statistical Analysis

The results were analyzed by using GraphPad Prism.9 and SPSS 19.0 software. Measurements are presented as the mean ± standard deviation. Differences between groups were analyzed by one-way ANOVA, and two-way comparisons were performed by *t*-test; statistical significance was set as α = 0.05.

## 3. Results

### 3.1. Reproductive System of Male Offspring

**Figure 3:**
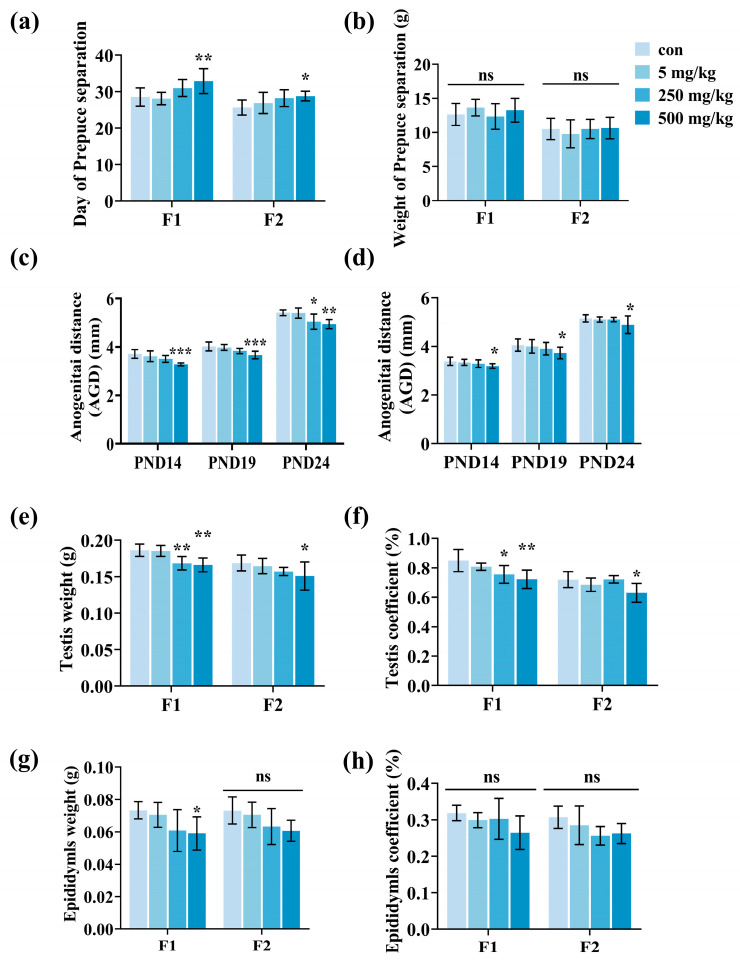
Reproductive system evaluation of male mice in F1 and F2 generations after paternal DEHP exposure. (**a**) F1 and F2 male foreskin separation time. (**b**) Weight of F1 and F2 male foreskin at separation. (**c**) Anogenital distances (mm) of F1 males PND14, 19, and 24. (**d**) Anogenital distances (mm) of F2 males PND14, 19, and 24. (**e**) Testis weight of F1 and F2 males. (**f**) Testicular coefficients for F1 and F2 males. (**g**) Epididymal weights of F1 and F2 males. (**h**) Epididymal coefficients for F1 and F2 males ((**a**–**d**), n = 9, (**e**–**h**), n = 6, *** *p* < 0.001, ** *p* < 0.01, * *p* < 0.05, and ns indicates no difference).

As illustrated in Figure 3a, the duration of foreskin separation in male mice from the F1 and F2 generations was extended as the exposure doses increased, with the most pronounced impact seen in the 500 mg/kg/d group (*p* < 0.05). Body weight at the time of foreskin separation did not change much between dose groups (Figure 3b). Anal–genital distances of male offspring were measured at postnatal days 14, 19, and 24, and showed a gradual decrease with increasing doses in male offspring (*p* < 0.05, Figure 3c,d).

The weights of reproductive organs were included in the evaluation of reproductive system injuries, with testicular and epididymal weights measured and organ coefficients calculated for both F1- and F2-generation male mice. It was observed that testis weight and organ coefficient decreased with increasing dose in male offspring (*p* < 0.05, Figure 3e,f). Moreover, although the weight of the epididymis decreased in F1 male mice, the organ coefficient did not exhibit any notable differences. In F2 male mice, both epididymis weight and organ coefficient tended to decrease, but again, no significant difference was noted (Figure 3g,h). These findings clearly indicate that paternal exposure to DEHP adversely impacts the development and maturation of the reproductive organ of male offspring.

### 3.2. Male Offspring Sperm Parameters

**Figure 4:**
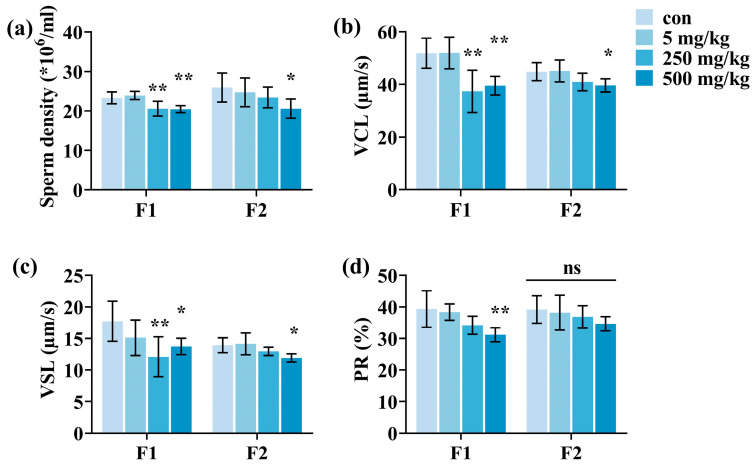
Evaluation of sperm quality in F1- and F2-generation male mice after paternal exposure to DEHP. (**a**) F1 and F2 male sperm density. (**b**) F1 and F2 male sperm curvilinear velocity (VCL). (**c**) F1 and F2 male sperm straight-line velocity (VSL). (**d**) F1 and F2 male percentage of spermatozoa with forward motile spermatozoa (PR%) (n = 6, ** *p* < 0.01, * *p* < 0.05, and ns indicates no difference).

To examine how paternal exposure to DEHP affects the sperm quality in male offspring, we assessed four sperm parameters: sperm density, curvilinear velocity (VCL), straight-line velocity (VSL), and percentage of forward movement (PR%). In Figure 4, the findings revealed a significant reduction in sperm density, VCL, VSL, and PR% with increasing doses in the F1 generation (*p* < 0.05). Even in the F2 generation, the sperm density along with VCL and VSL also diminished with escalating doses, with the most significant impacts noted in the 500 mg/kg/d group (*p* < 0.05). Conversely, there was no significant difference in PR% when compared to the control. This suggests that exposure of fathers to DEHP has a negative impact on the sperm quality of their male offspring.

### 3.3. Histology of Male Offspring Testes

**Figure 5:**
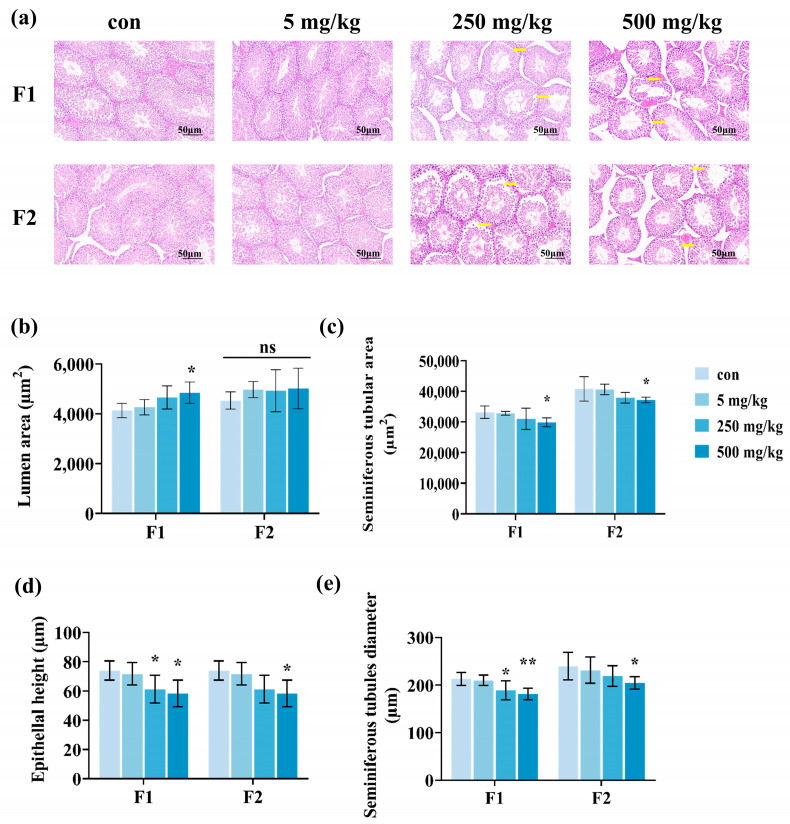
Testicular histology in F1- and F2-generation male mice after paternal exposure to DEHP. (**a**) Representative images of F1 and F2 male testis histology with a scale bar of 50 μM; yellow arrows indicate detachment of the spermatogenic epithelium. (**b**) Testicular lumen area in F1 and F2 males. (**c**) Testicular seminiferous tubular area in F1 and F2 males. (**d**) Epithelial height of spermatogenic tubules in F1 and F2 male testes. (**e**) Diameter of testicular seminiferous tubules in F1 and F2 males (n = 6, ** *p* < 0.01, * *p* < 0.05, and ns indicates no difference).

We examined the morphology of testicular tissues in male offspring. The testicular tissues of the male offspring in the DEHP-contaminated group exhibited disorganized spermatogenic tubules, vacuolization, detachment of the spermatogenic epithelium, breakage of the basement membrane, and other pathological damages. In contrast, the control group displayed intact spermatogenic epithelium and spermatogenic tubules arranged in an orderly manner (Figure 5a). Additionally, the lumen area within testicular tissues in the F1 generation demonstrated a notable increase as the dosage of the DEHP rose, while the increase in the lumen area for the F2 generation was not statistically significant (Figure 5b). The testicular tissue seminiferous tubular area, epithelial height, and diameter tended to decrease in the F1 and F2 generations, with a significant decrease in the high-dose group (*p* < 0.05, Figure 5c–e). Taken together, the results suggest that paternal exposure to DEHP significantly impairs the structure of the male testis in the offspring.

### 3.4. Male Offspring Reproductive Hormone Levels

**Figure 6:**
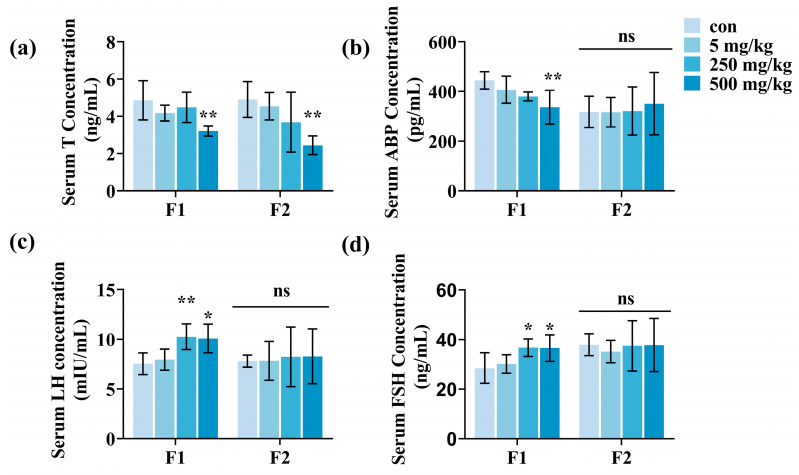
Changes in reproductive hormones in male offspring after paternal DEHP exposure. (**a**) Testosterone (T) levels in serum of F1 and F2 males. (**b**) Androgen-binding protein (ABP) levels in serum of F1 and F2 males. (**c**) Luteinizing hormone (LH) levels in serum of F1 and F2 males. (**d**) Follicle-stimulating hormone (FSH) levels in serum of F1 and F2 males (n = 5, ** *p* < 0.01, * *p* < 0.05, and ns indicates no difference).

Exposure to DEHP influences testosterone production. To explore the impact of paternal DEHP exposure on reproductive hormone levels in male offspring, we examined several important hormones. The findings indicated that testosterone levels exhibited a tendency to decrease in both generations of mice, with a notable reduction observed in the 500 mg/kg/d group (*p* < 0.05, Figure 6a). Androgen binding protein levels dropped in the F1 generation (*p* < 0.05, Figure 6b) and showed little variation in the F2 generation. In the F1 generation, levels of luteinizing hormone and follicle-stimulating hormone rose in 250 and 500 mg/kg/d groups (*p* < 0.05, Figure 6c,d), while remaining stable in the F2 generation.

### 3.5. Apoptosis in Male Offspring Testis Cells

**Figure 7:**
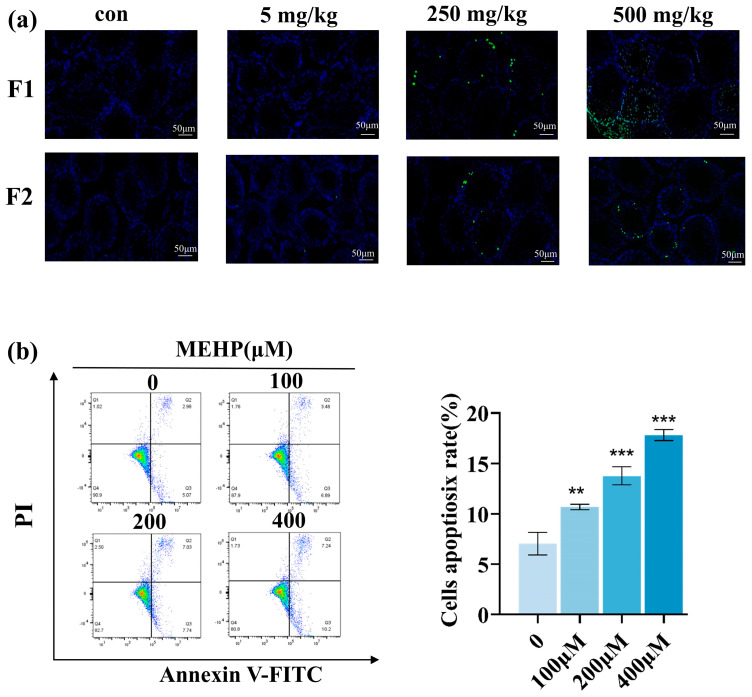
Testicular cell apoptosis in male offspring following paternal DEHP exposure. (**a**) TUNEL staining of F1 and F2 male testis tissues with a scale bar of 50 μM. TUNEL green fluorescent cells are apoptotic cells. (**b**) The apoptosis of TM3 cells treated with MEHP for 24 h was detected by flow cytometry. On the left is the representative flow scatter plot, and on the right is the percentage of apoptosis of TM3 cells (n = 3, *** *p* < 0.001, ** *p* < 0.01).

TUNEL staining (Figure 7a) revealed that male offspring showed an increased count of TUNEL-positive cells in the 250 and 500 mg/kg/d groups. This observation indicates that exposure of the parental to DEHP leads to apoptosis in the testicular cells of their offspring. MEHP is a metabolite of DEHP in vivo, which has a toxic effect on testes, and because of the continuous decrease in the testosterone level we chose TM3 cells for further experimental studies. Flow results showed that after 24 h of MEHP treatment of TM3 cells, the rate of apoptosis increased significantly with increasing dose (*p* < 0.05, Figure 7b), indicating that MEHP induced apoptosis in TM3 cells.

### 3.6. H3K27me3 Mediates DEHP Paternal Exposure-Induced Apoptosis in Male Offspring Testis Cells

**Figure 8:**
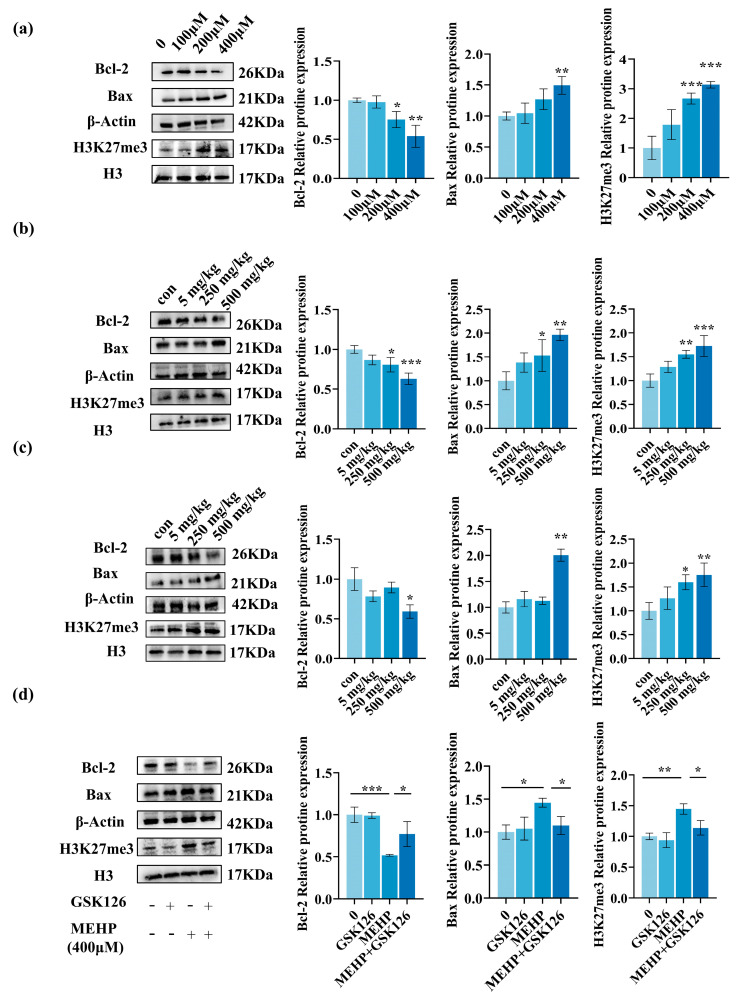
H3K27me3 mediates apoptosis in male offspring testis cells due to DEHP paternal exposure. (**a**) Immunoblot images and quantification of Bax, Bcl-2, and H3K27me3 levels in TM3 cells 24 h after MEHP treatment. (**b**) Immunoblot images and quantification of Bax, Bcl-2, and H3K27me3 levels in testicular tissue from F1 males. (**c**) Immunoblot images and quantification of Bax, Bcl-2, and H3K27me3 levels in testicular tissue from F2 males. (**d**) The “+” indicates treatment with MEHP/GSK126; “-” indicates no treatment. Immunoblot images and quantification of Bax, Bcl-2, and H3K27me3 after simultaneous treatment of TM3 cells with GSK126 inhibitor and MEHP for 24 h compared to MEHP-treated group (n = 3, *** *p* < 0.001, ** *p* < 0.01, * *p* < 0.05).

To further elucidate the occurrence of apoptosis at the molecular level in testicular cells following staining, Western blot analysis was conducted on animal and cell staining models. The results of the analysis show that the expression of Bax was elevated, while Bcl-2 expression was reduced after MEHP treatment of TM3 cells for 24 h (Figure 8a). Similarly, the protein Bax, which is linked to apoptosis, showed increased expression, and Bcl-2 exhibited decreased levels in the testicular tissues of male offspring following their fathers’ exposure to DEHP (Figure 8b,c). Furthermore, it has been established that the histone H3K27me3, an epigenetic modification, can inhibit Bcl-2 expression in response to environmental stressors, thereby promoting apoptosis. The findings of our study demonstrated an increase in H3K27me3 expression after 24 h of MEHP treatment in TM3 cells (Figure 8a), alongside heightened levels of H3K27me3 in the testicular tissues of male offspring (Figure 8b,c). To further elucidate the regulation of Bcl-2 by H3K27me3, we administered GSK126, an H3K27me3 inhibitor, to the TM3-treated group, effectively inhibiting the expression of H3K27me3 (Figure 8d). Subsequently, we observed a reduction in apoptosis, along with decreased expression of Bax and H3K27me3, and an increase in Bcl-2 expression (Figure 8d). The findings indicate that histone H3K27me3 is necessary for apoptosis in male offspring testis cells after paternal exposure to DEHP.

## 4. Discussion

DEHP is a well-known environmental endocrine disruptor that exerts detrimental effects on male reproductive health [25]. Numerous animal studies and analyses of population data have demonstrated that DEHP exposure leads to reproductive impairments [26,27]. Furthermore, exposure during pregnancy adversely impacts the growth, development, and fertility of offspring [28]. Studies involving Caenorhabditis elegans have shown that exposure to DEHP results in decreased offspring numbers and reproductive senescence across generations [29]. Nonetheless, the impacts and processes related to paternal exposure on male offspring have not been thoroughly examined. Thus, elucidating the effects of paternal DEHP exposure on male offspring and underlying mechanisms is essential. The concentration at which no adverse effects were observed for DEHP exposure in the EU risk assessment was 4.8 mg/kg/d. Additionally, SD rats exposed to 5 mg/kg/d DEHP did not result in adverse effects on testicular or developmental toxicity across three consecutive generations of male rats [30]. Conversely, subjecting pubertal male mice to a dosage of 250 mg/kg caused damage to testicular cells and disrupted normal testicular functions [31]. Additionally, decreased testosterone levels and hindered spermatogenesis has been found in the F1 male offspring of pregnancy exposure to a mixture of phthalates at 500 mg/kg [32]. In light of these results, we developed an experimental study that included a control group (corn oil) and exposure groups with DEHP doses of 5, 250, and 500 mg/kg/d.

The growth and development of offspring may indicate reproductive developmental toxicity resulting from paternal exposure. Anogenital distance (AGD) has been utilized as a marker for exposure to environmental endocrine disruptors, reflecting early androgenic effects and predicting reproductive health in adulthood [33]. Jorge’s study revealed that low-dose benzo(a)pyrene exposure in the rat father led to a significant decrease in AGD and reduced fertility in the F2 generation of male rats [34]. Furthermore, exposure to DEHP in mothers prior to conception has been linked to a delay in foreskin separation, a reduction in anogenital distance (AGD), and disrupted activation and functioning of the gonadotropic axis in male offspring [23]. Similarly, our study demonstrated that DEHP exposure resulted in delayed foreskin separation and decreased AGD in male mice across both F1 and F2 generations. Prepuce separation is a marker of the onset of male puberty, and the delay suggests that paternal exposure to DEHP causes delayed puberty in male offspring, which may be induced by antiandrogenic activity, as evidenced by the decline in AGD we detected [35]. Reproductive organ weight and sperm quality are critical indicators for assessing reproductive toxic effects in males, and direct exposure of male rats to DEHP has been shown to decrease sperm viability [8]. In this research, exposure of male mice to DEHP led to a decrease in both testicular and epididymal weights, along with decreased sperm quality in the F1 generation, and the weight loss of testis and epididymis and the decrease in sperm quality were still observed in the 500 mg/kg/d group of the F2 generation. This may be due to the fact that the H3K27me3 protein escapes epigenetic reprogramming events during transgenerational inheritance in the high-dose group, conferring conditions for the transmission of high-dose transgenerational reproductive damage [36]. Thus, partial reproductive impairment was still observed in the high-dose group. The difference in effects between the F2 and F1 generations may be due to the different intergenerational and transgenerational effects resulting from different exposure doses in the F0 generation [37]. Collectively, these data show that paternal exposure to DEHP contributes to reproductive damage in male offspring.

Normal endocrine function is crucial for male reproductive health. Our study showed that FSH and LH levels were elevated in the F1 generation, while testosterone levels were decreased in both the F1 and F2 generations. The results of the present study suggest that DEHP directly damages TM3 cells, resulting in decreased testosterone levels. The decrease in testosterone level activates the negative feedback regulation mechanism of the hypothalamic–pituitary–gonadal axis, which results in the upward elevation of FSH and LH levels secreted by the pituitary gland [38,39]. Testosterone promotes the formation of the male reproductive phenotype and facilitates spermatogenesis [32]. Given that TM3 cells are responsible for testosterone production, we opted to utilize the TM3 cell line in our in vitro studies. Numerous research efforts have shown that DEHP triggers apoptosis in testicular cells [40]. Our research revealed that paternal exposure to DEHP led to pathological harm in the offspring, which encompassed testicular tissue atrophy and TUNEL positivity observed in testicular cells, indicative of apoptosis. The result of changed expression levels of apoptosis-related proteins Bcl-2 and Bax within the testicular tissues of both F1 and F2 generations offer molecular support for the assertion that paternal DEHP exposure initiates testicular apoptosis in the progeny.

Exposure of parents to environmental endocrine disruptors leads to modifications in the epigenetic traits of all somatic cells derived from them by changing the epigenetic information in the germ cells, which subsequently affects the phenotype of their offspring [41]. H3K27me3 is a well-established epigenetic modification primarily associated with gene repression. Different cell types inhibit the expression of Bcl-2 anti-apoptotic molecules through high levels of H3K27me3 in the promoter region, which enhances the expression of the downstream pro-apoptotic molecule Bax, and finally induces cell apoptosis [42]. To explore the potential function of H3K27me3 in a transgenerational model of parental exposure to DEHP, we examined H3K27me3 expression levels in testicular tissues of the F1 and F2 generations, and showed that expression was elevated in both generations. Furthermore, we observed increased H3K27me3 expression and apoptosis in MEHP-treated TM3 cells after 24 h. The H3K27me3 protein level in the MEHP group was not completely restored after using the inhibitor GSK126, indicating that the GSK126 inhibitor could not completely inhibit the expression of the H3K27me3 protein. In addition to H3K27me3, Bcl-2 may also be affected by epigenetic modifications such as small noncoding RNAs and DNA methylation [43,44]. Thus, Bcl-2 protein levels were not completely reversed in the MEHP-stained group after addition of the inhibitor.

As research on the reproductive toxicity of DEHP progresses, the exposure doses selected in animal studies are increasingly reflective of actual human exposure levels in daily life. Nonetheless, it is important to evaluate the effects of long-term exposure of DEHP, particularly on transgenerational aftermath, due to its significant persistence and bioaccumulation in the environment. In this study we initially explored the role that histone methylation plays in a transgenerational model of paternal exposure to DEHP, without exploring more fully whether DNA methylation and small noncoding RNAs play a role in this, and how histone methylation is maintained. In the animal experiments, no intervention group was established concurrently, and the intervention experiments were ultimately conducted using an in vitro cellular model; we did not go further to investigate the regulation of Bcl-2 by H3K27me3 in our in vitro cellular experiments, and we will consider this work to enrich our study in the future. Our present study found that reproductive toxicity associated with paternal exposure to DEHP persisted into the F2 generation but the effect was not significant relative to the F1 generation; however, we did not conduct experiments with the F3 and F4 generations. Consequently, we cannot determine whether the reproductive system toxicity resulting from DEHP paternal exposure in male offspring recovers in subsequent generations and the mechanisms involved.

## 5. Conclusions

Our research indicated that exposure of fathers to DEHP leads to impaired growth and development, as well as reproductive dysfunction in male offspring. Furthermore, the modification of H3K27me3 is implicated as one of the epigenetic mechanisms contributing to offspring testicular cell apoptosis induced by paternal DEHP exposure. These findings indicate that reproductive system damage resulting from paternal DEHP exposure can be transmitted across generations, extending to the F2 generation, and has long-term implications for reproductive health and fertility in animals. This research deepens our comprehension of the negative impacts of DEHP exposure from fathers on their male offspring. Additionally, it may provide a foundation for future studies on reproductive toxicity and the underlying mechanisms affecting male offspring due to paternal DEHP exposure.

## Data Availability

The data used to support the findings of this study are available from the corresponding author upon reasonable request.
